# Updates on the controversial roles of regulatory lymphoid cells in idiopathic pulmonary fibrosis

**DOI:** 10.3389/fimmu.2024.1466901

**Published:** 2024-09-25

**Authors:** Anna V. Curioni, Raphaël Borie, Bruno Crestani, Doumet Georges Helou

**Affiliations:** ^1^ Université Paris Cité, Institut national de la santé et de la recherche médicale (INSERM), Physiopathologie et épidémiologie des maladies respiratoires (PHERE), Paris, France; ^2^ Service Pneumologie A, Assistance publique – Hôpitaux de Paris (AP-HP), Hôpital Bichat, Paris, France

**Keywords:** pulmonary fibrosis, lung inflammation, immune regulation, regulatory T cells, regulatory B cells

## Abstract

Idiopathic pulmonary fibrosis (IPF) is the most common and severe form of pulmonary fibrosis, characterized by scar formation in the lung interstitium. Transforming growth factor beta (TGF-β) is known as a key mediator in the fibrotic process, acting on fibroblasts and mediating their proliferation and differentiation into myofibroblasts. Although the immune system is not considered responsible for the initiation of IPF, markers of tolerogenic immunity define the pro-fibrotic microenvironment in the lungs. In homeostatic conditions, regulatory T cells (Tregs) constitute the main lymphoid population responsible for maintaining peripheral tolerance. Similar to Tregs, regulatory B cells (Bregs) represent a recently described subset of B lymphocytes with immunosuppressive functions. In the context of IPF, numerous studies have suggested a role for Tregs in enhancing fibrosis, mainly via the secretion of TGF-β. In humans, most studies show increased percentages of Tregs associated with the severity of IPF, although their exact role remains unclear. In mice, the most commonly used model involves triggering acute lung inflammation with bleomycin, leading to a subsequent fibrotic process. Consequently, data are still conflicting, as Tregs may play a protective role during the inflammatory phase and a deleterious role during the fibrotic phase. Bregs have been less studied in the context of IPF, but their role appears to be protective in experimental models of lung fibrosis. This review presents the latest updates on studies exploring the implication of regulatory lymphoid cells in IPF and compares the different approaches to better understand the origins of conflicting findings.

## 1 Introduction

IPF is a progressive interstitial lung disease (ILD) characterized by thickening and scarring of lung parenchyma (1). It histologically presents as usual interstitial pneumonia (UIP) (2), with symptoms related to impaired gas exchange, including dyspnea, dry cough, and fatigue (3). The prognosis is poor, with a median survival of 3-5 years after diagnosis (4). Dominant hypotheses suggest that IPF results from genetic predisposition added to external factors, such as smoking, pollution, and viral infections, mainly in elderly males (5). Repetitive epithelial injury in the alveoli leads to an abnormal healing process involving senescence, epigenetic reprogramming, aberrant epithelial-mesenchymal crosstalk, and inflammatory cell recruitment (6–8). Fibroblasts and recruited fibrocytes differentiate within the injured areas into myofibroblasts, which become resistant to apoptosis, proliferate, and exhibit uncontrolled production of extracellular matrix (ECM) components, notably collagen (9). Resident and recruited immune cells can polarize towards a pro-fibrotic phenotype and directly enhance the accumulation of ECM via the secretion of pro-fibrotic mediators, such as TGF-β, interleukin (IL-) 10, tumor necrosis factor (TNF), and matrix metalloproteinases (MMP) (5). Current treatment relies on nintedanib and pirfenidone, two anti-fibrotic drugs (10). Pirfenidone slows collagen deposition and suppresses TGF-β activity (2), but its exact mechanism of action has not yet been fully elucidated (10). Nintedanib is a tyrosine kinase inhibitor that downregulates the production of ECM proteins and inhibits TGF-β-induced myofibroblast differentiation (11). Both drugs have significant adverse effects and fail to stop the progression of the disease toward its fatal outcome (12). For the time being, lung transplantation remains the only curative treatment (2), therefore, a deeper understanding of the mechanisms underlying IPF initiation and development is fundamental to unveil targeted and efficient therapies.

For years, the hypothesis of the inflammatory origin of IPF has seen an exploration of different immune cells and mediators (13). However, the non-response of IPF patients to immunosuppressive therapeutic strategies has reduced the interest in targeting the immune system. The fatal blow to this hypothesis came from the PANTHER-IPF trial (14). A combination of prednisone, azathioprine, and N-acetylcysteine resulted in increased risks of death and hospitalization in IPF patients. The failure of these trials not only suggests a limited secondary role for inflammation in IPF but also hints at the immune system potentially playing a critical regulatory role in this disease. This could explain the detrimental outcomes observed with its suppression. Therefore, instead of outright suppression, modulating the immune system may prove beneficial in managing IPF (15). For most myeloid immune cell subsets, namely neutrophils, macrophages, fibrocytes, and monocytes, a pro-fibrotic role has been reported. Concerning the adaptive immune response, T and B lymphocytes may play a protective or detrimental role depending on the subpopulation involved (16).

Among these subpopulations, regulatory B and T cells, namely Bregs and Tregs, are crucial in dampening the immune response and preserving immune homeostasis (17, 18). Aberrant activation of these subpopulations might be implicated in fibrotic conditions. While a wide amount of work exists on the role of Tregs in IPF, although contradictory, Bregs have not yet been extensively studied and appear to have a protective role. This review summarizes the conflicting findings from the past five years regarding the roles of Treg and Breg subsets in IPF development.

## 2 Regulatory T cells

Tregs are mainly identified as CD4+ CD25+ FoxP3+ T cells and are known to be essential in maintaining peripheral immune tolerance, thereby preventing aberrant activation of the immune system and the development of autoreactive cells (17). Once activated, Tregs are a major source of immunosuppressive cytokines, mainly IL-10, TGF-β, and IL-35, that limit the differentiation, proliferation, and activation of effector CD4+ T cells. Although Treg activation is antigen-specific, bystander suppression could be exerted on neighboring T cells independently of antigens (19). Other direct mechanisms to suppress effector T cells were described, including the blockade of dendritic cell (DC) maturation, either through lymphocyte-activation gene 3 binding to the major histocompatibility class II molecules, or through cytotoxic T-lymphocyte antigen 4 (CTLA4)-CD80/CD86 interactions. This leads to the secretion of indoleamine 2,3-dioxygenase and consequent inhibition of DCs’ activity (20).

Given the crucial role of Tregs in dampening the immune response and thereby maintaining homeostasis, dysregulation of their activity can result in various diseases, such as autoimmune diseases, allergies, cancer, and chronic infections (21). Though studies remain controversial, Tregs are also implicated in various fibrotic conditions, such as hepatic fibrosis (22) and systemic sclerosis (23).

### 2.1 Tregs in murine models of pulmonary fibrosis

**Figure 1:**
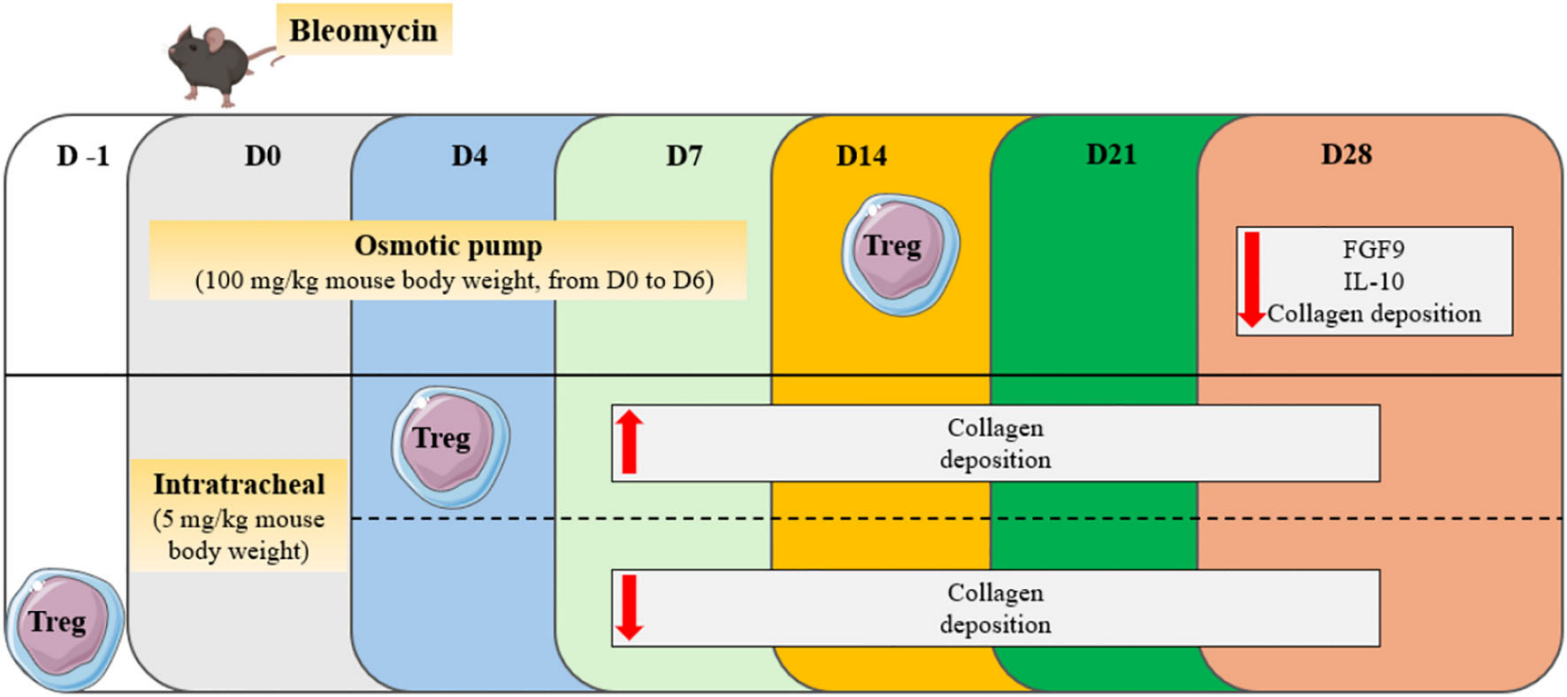
Effect of Treg transfer in the bleomycin model of PF. Depending on the adoptive transfer day (D-1, D4, D14), Tregs can play a protective or detrimental role across the different phases of bleomycin-induced lung injury.

The secretion of IL-10 and TGF-β by Tregs would have a deleterious effect on the mechanisms involved in the pathophysiology of PF (24). However, it appears that depending on their suppressive phenotype and the stage of the disease, regulatory T cells could also play a protective role in both human and murine models of PF (25–27). The bleomycin model is widely used in mice studies. It is characterized by two distinct phases: the inflammatory phase usually occurs during the first week after bleomycin injection, while the fibrotic phase takes place starting from the second week. In a study conducted by Takao et al., PF was induced in mice through oropharyngeal administration of bleomycin, and it was demonstrated that human bone marrow-derived serum-free mesenchymal stromal cells (SF−MSCs) administrated intravenously 4 days after bleomycin treatment have a beneficial effect on the disease. This protective role is exerted through the induction of regulatory T cells, whose numbers are increased in the blood and lungs of mice on the 2^nd^ day after SF−MSCs treatment. Indeed, in blood and lungs, SF−MSCs lead to increased TGF-β1 and decreased IL-6 levels at day 7 and 14, respectively. Consistently, Treg depletion causes a loss of the antifibrotic effects of SF-MSCs (26). Furthermore, it was demonstrated in mice that the adoptive transfer of Tregs 14 days after bleomycin treatment, reduces IL-10 and FGF9 levels in plasma, and subsequently decreases fibrotic lesions at day 28 (25). Given the established link between the aryl hydrocarbon receptor (AhR) activation and Treg generation (28), Takei et al. demonstrated that AhR engagement after bleomycin administration increases the number of pulmonary Tregs and alleviates fibrosis development (27). Zhang and colleagues showed that from days 3 to 28 after bleomycin instillation, Treg percentages are increased in both peripheral blood and spleen. Additionally, the proportion in the blood is positively correlated with the fibrotic score. From day 3 to day 14, Th1 responses are prevalent, while a switch towards Th2 polarization starts from day 14. Tregs participate in this Th2 polarization, as their co-culture with spleen lymphocytes leads to decreased interferon (IFN-) γ and increased IL-4 secretion. Additionally, the adoptive transfer of Tregs in bleomycin-treated mice during the inflammatory phase worsens fibrotic lesions, while preventive transfer leads to decreased hydroxyproline (HP) content in the lungs (29) (**Figure 1**). When PF is induced in mice through overexpression of TGF-β, the levels of regulatory T cells in the lungs and pro-fibrotic markers correlate with the tissular sodium level, a detrimental factor in a fibrotic environment (30). In a recent study, Liu et al. showed that the percentage of Tregs from CD4+ T lymphocytes increases from day 3 to day 14 after bleomycin administration, while it decreases from day 14 to day 28. Also, they showed that anti-fibrotic drugs such as pirfenidone, prednisolone, neferine, and isoliensinine, can alter Treg dynamics across the different phases of lung injury, highlighting therefore a new factor that could explain data variabilities across the different studies (31). Interestingly, Zhao et al. showed that intermittent fasting attenuates bleomycin-induced PF and decreases the percentages and counts of pulmonary Tregs. The authors therefore suggested that the reduction of Tregs through intermittent fasting dampens PF development (32).

In the context of the acute exacerbation (AE) of PF, Tregs’ role remains also controversial. In a pulmonary fibrosis model induced by adenoviral vector-mediated overexpression of TGF-β1 (AdTGF-β1) and exacerbated with *S. pneumoniae* infection, Treg numbers were shown to be significantly increased in both bronchoalveolar lavage (BAL) and lungs at day 14 compared to days 7 and 21. While depletion of Tregs worsens the exacerbation, expansion with IL-2/anti–IL-2 monoclonal antibody (mAb) complexes leads to its inhibition. The levels of TNFα, IL-6, and TGF-β1 are decreased 14 days after the injection of mAb complexes, suggesting that Tregs modulate T cell cytokine responses and dampen PF development (33). Contrarily, Tao et al. showed that Tregs are involved in the macrophage inducible C-type lectin (Mincle)-mediated AE of PF in the bleomycin mouse model. Mincle deficiency results in a smaller Treg population in BAL, improved survival, and milder fibrotic lesions. Thus, the authors propose a detrimental role for Tregs in the AE of PF (34).

### 2.2 Tregs in IPF patients

Although mechanistic data concerning the role of human Tregs in IPF in the last 5 years seems to be lacking, the reported proportions in patients compared to controls are not always consistent from one study to another. In particular, peripheral blood, BAL, and lung tissue do not always follow a common thread among IPF patients, and inconsistency is also encountered among samples of the same nature. A protective role for Tregs in humans has been suggested and appears to be tightly regulated by the expression of immune receptors. Decreased surface expression of the inducible T-cell costimulator (ICOS) on CD4+ T lymphocytes, a receptor responsible for the maintenance of immunosuppressive Tregs, is significantly correlated with decreased lung function, while a high expression results in improved survival (35). Conversely, Programmed cell death protein 1 (PD-1) blockade on myofibroblasts leads to increased Treg differentiation and decreased collagen levels, suggesting a protective role for this T cell population in IPF (36). Yin et al. used a risk assessment model of inflammation-linked genes to distinguish two groups of IPF patients based on their overall survival. Cell infiltration analysis revealed that the infiltration of Tregs is higher in the BAL of the high-risk group as compared to the low-risk group (37). Percentages from CD4+ T cells are also higher in BAL of IPF patients compared to sarcoidosis patients (38) and connective tissue disease-associated ILDs (39). Percentages of Tregs from blood CD4+ T cells are instead lower in IPF than in sarcoidosis patients (38). Compared to healthy controls, some authors reported that the percentages of Tregs from CD4+ T cells in the blood of IPF patients are higher (40), while others showed a decreased percentage (36). Moreover, it seems that activated fibroblasts do not enhance the Treg population, as co-culture of TGF-β-stimulated human fibroblasts with CD4+ T cells does not cause them to differentiate into Tregs (36). The recruitment of Tregs in IPF patients compared to healthy controls was also assessed by Wu et al. using publicly available datasets. The infiltration status of 22 types of immune cells was analyzed using a CIBERSORT algorithm (41). While some studies report no difference between Treg cell percentages in lungs from IPF patients compared to controls (42), Dai et al. found decreased percentages of Tregs in IPF tissues (43). A recent study by Unterman et al., based on transcriptomic data, demonstrated different data in the blood (44). Single-cell RNA sequencing was performed on peripheral blood mononuclear cells (PBMCs) from healthy subjects and stable or progressive IPF. It was shown that Tregs are increased in PBMCs from patients presenting with the progressive form of the disease compared to the stable group, and in combined IPF patients compared to controls. However, no difference was noticed between stable IPF patients and healthy controls. The authors therefore proposed that the discrepancy in Treg variation reported by the literature may be due to a lack of distinction between patients at different stages of the disease. Results also suggested that Treg recruitment implicates the interactions of the receptors CCR4 and CCR8 on Tregs with their respective ligands CCL22 and CCL18. Both chemokines are known to be secreted by DCs and macrophages and show higher levels in plasma from IPF patients compared to controls. Moreover, Tregs are the only lymphocyte population that increases in the lungs (44). In murine models of PF, the fibrotic response originates from strong inflammatory processes that develop following bleomycin injection (45). It is therefore coherent that Tregs may be protective at least during the inflammatory phase but then switch roles to contribute to fibrosis by secreting pro-fibrotic mediators. However, the cause of human IPF is unknown, and it remains unclear whether inflammation participates in the initiation of the disease or is a collateral consequence of a fatal pathophysiological process. Therefore, an understanding of how Tregs mechanistically participate in IPF development is still lacking, especially since the intrinsic immunosuppressive role of Tregs might not be sufficient to point out their pro-fibrotic contribution.

## 3 Bregs in PF

Besides their classical functions, B cells and plasmablasts can differentiate into regulatory B cells. Induction of these cells can occur following stimulation with LPS, cytokines such as IL-1β, IL-2, IL-6, and IFN-α, or contact with T cells. Plasmacytoid DCs have also been demonstrated to induce the differentiation of B cells into Bregs through binding of CD40 and secretion of IFN-α. Additionally, Immunoglobulin-A, PD-L1, TIGIT, and Fas-L expressing Bregs can be generated through the binding of proliferation-inducing ligand (APRIL) to naive B cells (46). Bregs secrete IL-10, IL-35, and TGF-β (47), and can express PD-1 ligand, Granzyme B, CD39, CD73, and the AhR (46), thereby contributing to the dampening of the immune reaction to prevent chronic inflammation and the development of autoimmune conditions (47). Indeed, Bregs inhibit the differentiation of pro-inflammatory lymphocytes, such as Th1, Th17, and CD8+ T cells, while promoting the activity of immunosuppressive Tregs and inducible NK cells (47). The phenotypic characterization of Bregs remains debatable, even though secretion of IL-10 has been commonly referred to as the “hallmark of Breg suppression” for years (48). Although the involvement of IL-10 in the pathophysiology of IPF is well known, it being a pro-fibrotic cytokine, the role of Bregs remains yet to be clarified, with works mainly focusing on quantitative assessments in patients’ samples. A study by Asai et al. revealed that the proportion of Bregs in blood among total CD3- CD19+ B cells is lower in IPF patients compared to healthy controls, with lower numbers in patients presenting a more severe form of the disease. This suggests a protective role of Bregs in this context (49). D’Alessandro et al. also described lower percentages of Bregs from total B cells in the peripheral blood of IPF patients compared to the healthy control group. Interestingly, nintedanib treatment can restore Breg numbers and consequently redirect the inflammatory responses (50). Another study reported that in the bleomycin or adenoviral TGF-β-induced models of pulmonary fibrosis, Bregs are less than 1% of total B cells. In both models, Breg percentages from total B cells are higher in fibrotic lungs than in controls, but B cell depletion does not affect fibrosis development (51). Breg’s intrinsic immunosuppressive role may suggest novel perspectives regarding the modulation of the immune response in IPF. Therefore, their involvement in IPF deserves to be further elucidated.

## 4 Discussion

While Bregs are relatively new to the field of IPF research, the role of Tregs in this pathology remains a topic of considerable debate, and robust strategies targeting these populations have yet to be developed. Most human studies focus on variations in Treg percentages in the blood and BAL, resulting in inconsistent findings (**Table 1**). Many factors might underlie this discrepancy starting with the heterogeneity of the disease itself and the different stages of progression. Indeed, various mechanisms, which may differ from patient to patient, could initiate IPF and uniquely affect the balance between immune cell populations (52, 53). To partly address this challenge, a thorough stratification strategy of IPF patients according to various epidemiological and clinical parameters might be essential to detect variations between subgroups. This aligns with the recent study by Unterman et al., which underscores the importance of considering the disease’s progressive nature when assessing Treg quantitative variations (44). Additionally, basic demographic factors like gender and age are not to be neglected in the stratification strategy. It is known that males generally have more Tregs than females and that natural Tregs accumulate with age. Therefore, these factors should be considered when assessing variabilities across studies. Since older males have a high IPF prevalence, it is tempting to speculate that Treg accumulation in the lungs increases the risk of developing IPF.

**Table 1:** Variations in the Treg and Breg populations in murine models of PF and IPF patients.

MOUSE					
Reference	Model of PF/Species	Treg variation	Specimen	Effect on fibrosis development	Treg role
(26)	Bleomycin mouse model – SF-MSCs administration at D4	Increased counts	Blood and lungs D6	Antifibrotic effect of SF-MSCs exerted through Treg induction. Increased TGFβ at D7, decreased IL-6 at D14	Protective
(25)	Bleomycin mouse model – Treg transfer on D14			Reduced fibrotic lesions. Decreased IL-10 and FGF9 in plasma at D9	Protective
(27)	Bleomycin mouse model – AhR ligand treatment at D0, D1, D2	Increased counts	Lungs	Dampened inflammatory phase and thereby fibrosis	Protective
(29)	Bleomycin mouse model	Increased percentages	Peripheral blood and spleen	Positive correlation with the fibrotic score	Deleterious
		-adoptive transfer of Tregs during the inflammatory phase		Worsening of the fibrotic lesions	Deleterious
		-preventive transfer of Tregs		Decreased hydroxyproline content in the lungs	Protective
(30)	TGF-β1 transgenic mouse model	Increased percentages	Lungs	Positive correlation with Na+ levels in the tissue	Deleterious
(31)	Bleomycin mouse model	Increased percentages (day 3 to 14)	Peripheral blood		
		Decreased percentages (day 14 to 28)			
(32)	Bleomycin mouse model - intermittent fasting	Decreased counts and percentages	Lungs	PF attenuation	Protective
(33)	AdTGF-β1 mouse model, *S. pneumoniae-*induced exacerbation	Increased counts, with the peak on D14	Lungs and BAL		Protective
		Depletion		Worsened exacerbation	
		Expansion		Inhibited exacerbation	
(34)	Bleomycin mouse model, Mincle-mediated AE	Decreased counts following the silencing of Mincle-coding gene	BAL	Improved survival and milder fibrotic lesions	Deleterious
Reference	Model of PF		Breg variation		Specimen
(51)	Bleomycin/AdTGFβ1 mouse model		Higher percentages		Lung tissue
HUMAN					
Reference	Treg variation		Specimen		Control
(37)	Increased percentages in high-risk patients		BAL		Low-risk group
(38)	Increased percentages				Sarcoidosis
	Decreased percentages		Peripheral blood		
	Increased percentages		BAL		CTD-ILDs
(40)	Increased percentages		Peripheral blood		Healthy
(36)	Decreased percentages				
(42)	No difference		Lungs		Non-fibrotic
(43)	Decreased percentages				
Reference	Breg variation		Specimen		Control
(49)	Decreased % from total B cells		Peripheral blood		Healthy
(50)	Decreased % from total B cells, restored with Nintedanib		Peripheral blood		Healthy

Another critical point is the heterogeneity of Tregs themselves and their potential plasticity in IPF. Various Treg subpopulations may emerge and diminish throughout the disease progression, making it less informative to focus solely on total Treg numbers. Instead, examining distinct Treg profiles at different stages and severity levels of IPF might provide more valuable insights into the potential existence of pro- and anti-fibrotic subsets of Tregs in IPF. For instance, different Treg subsets can be identified according to their chemokine receptor expression, such as CXCR3+ Tregs and CCR6+ Tregs, highlighting their functional diversity. More transcriptomic studies will also be required to understand the specific gene expression patterns associated with Treg subsets and their functions in IPF.

Beyond understanding the global protective or deleterious role of Tregs in IPF, it is crucial to determine if these cells are antigen-specific and activated to increase tolerance toward specific antigens. Identifying the self- or non-self antigens that trigger Treg recruitment to the lungs can lead us to a better investigation of the factors triggering IPF development. If the activation is antigen-independent, understanding the mechanisms behind this activation is equally important.

In mice, studying Tregs in the context of bleomycin-induced models presents significant challenges. Unlike bleomycin-induced lung injury, where fibrosis occurs as a consequence of acute lung inflammation, IPF is characterized by chronic and progressive scarring of lung tissue. This fundamental difference undermines the utility of bleomycin models for accurately studying the role of Tregs in IPF. In particular, depletion and adoptive transfer experiments do not account for the plasticity and heterogeneity of Tregs across the disease stages.

While novel technologies have revolutionized research in the field of immunology, the study of Tregs and Bregs in IPF still has much ground to cover and warrants deeper investigation into its underlying mechanisms. However, it’s important to acknowledge the many limitations, such as the difficulty in accessing human samples and the relatively limited numbers of these cell populations. Additionally, the lack of relevance of mouse models to human disease poses further challenges to achieving a comprehensive understanding. Moving forward, addressing these challenges with collaborative efforts and innovative approaches could be beneficial for improving therapeutic outcomes for IPF patients.

## References

[1] Richeldi L Collard HR Jones MG. Idiopathic pulmonary fibrosis. Lancet. (2017) 389:1941–52. doi: 10.1016/S0140-6736(17)30866-8 28365056

[2] Glass DS Grossfeld D Renna HA Agarwala P Spiegler P DeLeon J. Idiopathic pulmonary fibrosis: Current and future treatment. Clin Respir J. (2022) 16:84–96. doi: 10.1111/crj.13466 35001525 PMC9060042

[3] Bloem AEM Houben-Wilke S Mostard RLM Stoot N Janssen DJA Franssen FME. Respiratory and non-respiratory symptoms in patients with IPF or sarcoidosis and controls. Heart Lung. (2023) 61:136–46. doi: 10.1016/j.hrtlng.2023.05.013 37269615

[4] Luppi F Kalluri M Faverio P Kreuter M Ferrara G. Idiopathic pulmonary fibrosis beyond the lung: understanding disease mechanisms to improve diagnosis and management. Respir Res. (2021) 22:109. doi: 10.1186/s12931-021-01711-1 33865386 PMC8052779

[5] Phan THG Paliogiannis P Nasrallah GK Giordo R Eid AH Fois AG. Emerging cellular and molecular determinants of idiopathic pulmonary fibrosis. Cell Mol Life Sci. (2021) 78:2031–57. doi: 10.1007/s00018-020-03693-7 PMC7669490 33201251

[6] Moss BJ Ryter SW Rosas IO. Pathogenic mechanisms underlying idiopathic pulmonary fibrosis. Annu Rev Pathol Mech Dis. (2022) 17:515–46. doi: 10.1146/annurev-pathol-042320-030240 34813355

[7] Froidure A Marchal-Duval E Homps-Legrand M Ghanem M Justet A Crestani B. Chaotic activation of developmental signalling pathways drives idiopathic pulmonary fibrosis. Eur Respir Rev Off J Eur Respir Soc. (2020) 29:190140. doi: 10.1183/16000617.0140-2019 PMC9488512 33208483

[8] Ghanem M Archer G Crestani B Mailleux AA. The endocrine FGFs axis: A systemic anti-fibrotic response that could prevent pulmonary fibrogenesis? Pharmacol Ther. (2024) 259:108669. doi: 10.1016/j.pharmthera.2024.108669 38795981

[9] Mei Q Liu Z Zuo H Yang Z Qu J. Idiopathic pulmonary fibrosis: An update on pathogenesis. Front Pharmacol. (2022) 12:797292. doi: 10.3389/fphar.2021.797292 35126134 PMC8807692

[10] Heukels P Moor CC von der Thüsen JH Wijsenbeek MS Kool M. Inflammation and immunity in IPF pathogenesis and treatment. Respir Med. (2019) 147:79–91. doi: 10.1016/j.rmed.2018.12.015 30704705

[11] Rangarajan S Kurundkar A Kurundkar D Bernard K Sanders YY Ding Q. Novel mechanisms for the antifibrotic action of nintedanib. Am J Respir Cell Mol Biol. (2016) 54:51–9. doi: 10.1165/rcmb.2014-0445OC PMC4742925 26072676

[12] Selvarajah B Platé M Chambers RC. Pulmonary fibrosis: Emerging diagnostic and therapeutic strategies. Mol Aspects Med. (2023) 94:101227. doi: 10.1016/j.mam.2023.101227 38000335

[13] Martinez FJ Collard HR Pardo A Raghu G Richeldi L Selman M. Idiopathic pulmonary fibrosis. Nat Rev Dis Primer. (2017) 3:17074. doi: 10.1038/nrdp.2017.74 29052582

[14] Prednisone, azathioprine, and N-acetylcysteine for pulmonary fibrosis. N Engl J Med. (2012) 366:1968–77. doi: 10.1056/NEJMoa1113354 PMC3422642 22607134

[15] Pokhreal D Crestani B Helou DG. Macrophage implication in IPF: Updates on immune, epigenetic, and metabolic pathways. Cells. (2023) 12:2193. doi: 10.3390/cells12172193 37681924 PMC10486697

[16] Deng L Huang T Zhang L. T cells in idiopathic pulmonary fibrosis: crucial but controversial. Cell Death Discov. (2023) 9:1–6. doi: 10.1038/s41420-023-01344-x 36788232 PMC9929223

[17] Shevyrev D Tereshchenko V. Treg heterogeneity, function, and homeostasis. Front Immunol. (2020) 10:3100. doi: 10.3389/fimmu.2019.03100 31993063 PMC6971100

[18] Catalán D Mansilla MA Ferrier A Soto L Oleinika K Aguillón JC. Immunosuppressive mechanisms of regulatory B Cells. Front Immunol. (2021) 12:611795. doi: 10.3389/fimmu.2021.611795 33995344 PMC8118522

[19] Kumar P Arbieva ZH Maienschein-Cline M Ganesh BB Ramasamy S Prabhakar BS. Induction of antigen-independent proliferation of regulatory T-Cells by TNF superfamily ligands OX40L and GITRL. Methods Mol Biol Clifton NJ. (2021) 2248:63–71. doi: 10.1007/978-1-0716-1130-2_4 33185867

[20] Rahman T MdF S Casellato A. Dissecting emerging aspects of regulatory circuitry in man and mice: Regulatory T cell biology. Adv Biosci Biotechnol. (2018) 09:443–68. doi: 10.4236/abb.2018.99031

[21] Cheru N Hafler DA Sumida TS. Regulatory T cells in peripheral tissue tolerance and diseases. Front Immunol. (2023) 14:1154575. doi: 10.3389/fimmu.2023.1154575 37197653 PMC10183596

[22] Savage TM Fortson KT de Los Santos-Alexis K Oliveras-Alsina A Rouanne M Rae SS. Amphiregulin from regulatory T cells promotes liver fibrosis and insulin resistance in non-alcoholic steatohepatitis. Immunity. (2024) 57:303–318.e6. doi: 10.1016/j.immuni.2024.01.009 38309273 PMC10922825

[23] Cheon SY Park JH Ameri AH Lee RT Nazarian RM Demehri S. IL-33/Regulatory T-Cell axis suppresses skin fibrosis. J Invest Dermatol. (2022) 142:2668–2676.e4. doi: 10.1016/j.jid.2022.03.009 35341735 PMC9511765

[24] Boveda-Ruiz D D’Alessandro-Gabazza CN Toda M Takagi T Naito M Matsushima Y. Differential role of regulatory T cells in early and late stages of pulmonary fibrosis. Immunobiology. (2013) 218:245–54. doi: 10.1016/j.imbio.2012.05.020 22739236

[25] Kamio K Azuma A Matsuda K Usuki J Inomata M Morinaga A. Resolution of bleomycin-induced murine pulmonary fibrosis *via* a splenic lymphocyte subpopulation. Respir Res. (2018) 19:71. doi: 10.1186/s12931-018-0783-2 29690905 PMC5978999

[26] Takao S Nakashima T Masuda T Namba M Sakamoto S Yamaguchi K. Human bone marrow-derived mesenchymal stromal cells cultured in serum-free media demonstrate enhanced antifibrotic abilities *via* prolonged survival and robust regulatory T cell induction in murine bleomycin-induced pulmonary fibrosis. Stem Cell Res Ther. (2021) 12:506. doi: 10.1186/s13287-021-02574-5 34530920 PMC8444523

[27] Takei H Yasuoka H Yoshimoto K Takeuchi T. Aryl hydrocarbon receptor signals attenuate lung fibrosis in the bleomycin-induced mouse model for pulmonary fibrosis through increase of regulatory T cells. Arthritis Res Ther. (2020) 22:20. doi: 10.1186/s13075-020-2112-7 32033616 PMC7006193

[28] Zhang Q Zhu Y Lv C Fang Y Liao M Xia Y. AhR activation promotes treg cell generation by enhancing Lkb1-mediated fatty acid oxidation *via* the Skp2/K63-ubiquitination pathway. Immunology. (2023) 169:412–30. doi: 10.1111/imm.13638 36930164

[29] Zhang J-H Deng J-H Yao X-L Wang J-L Xiao J-H. CD4+CD25+ tregs as dependent factor in the course of bleomycin-induced pulmonary fibrosis in mice. Exp Cell Res. (2020) 386:111700. doi: 10.1016/j.yexcr.2019.111700 31678213

[30] D’Alessandro-Gabazza CN Kobayashi T Yasuma T Toda M Kim H Fujimoto H. A staphylococcus pro-apoptotic peptide induces acute exacerbation of pulmonary fibrosis. Nat Commun. (2020) 11:1539. doi: 10.1038/s41467-020-15344-3 32210242 PMC7093394

[31] Liu W Zhang J-H Gao L Xiao J-H. Correlation between the dynamic changes of γδT cells, Th17 cells, CD4+CD25+ regulatory T cells in peripheral blood and pharmacological interventions against bleomycin-induced pulmonary fibrosis progression in mice. Exp Cell Res. (2024) 439:114098. doi: 10.1016/j.yexcr.2024.114098 38796136

[32] Zhao Y Yang J Zhang Q Chen X Liang W Zheng Y. Fasting alleviates bleomycin-induced lung inflammation and fibrosis *via* decreased tregs and monocytes. Adv Med Sci. (2024) 69:303–11. doi: 10.1016/j.advms.2024.07.004 38986767

[33] Moyé S Bormann T Maus R Sparwasser T Sandrock I Prinz I. Regulatory T cells limit pneumococcus-induced exacerbation of lung fibrosis in mice. J Immunol. (2020) 204:2429–38. doi: 10.4049/jimmunol.1900980 32213566

[34] Tao C Xian H Nian-yu Z Jia-cui S Dong W Hui-ping L. C-type lectin mincle initiates IL-17-mediated inflammation in acute exacerbations of idiopathic pulmonary fibrosis. BioMed Pharmacother. (2023) 159:114253. doi: 10.1016/j.biopha.2023.114253 36680813

[35] Bonham CA Hrusch CL Blaine KM Manns ST Vij R Oldham JM. T cell co-stimulatory molecules ICOS and CD28 stratify idiopathic pulmonary fibrosis survival. Respir Med X. (2019) 1:100002. doi: 10.1016/j.yrmex.2019.100002 32455343 PMC7243672

[36] Wang B Bai W Ma H Li F. Regulatory effect of PD1/PD-ligand 1 (PD-L1) on treg cells in patients with idiopathic pulmonary fibrosis. Med Sci Monit. (2020) 26. doi: 10.12659/MSM.927577 PMC7786833 33386384

[37] Yin Y-Q Peng F Situ H-J Xie J-L Tan L Wei J. Construction of prediction model of inflammation related genes in idiopathic pulmonary fibrosis and its correlation with immune microenvironment. Front Immunol. (2022) 13:1010345. doi: 10.3389/fimmu.2022.1010345 36601116 PMC9806212

[38] Zhang H Jiang D Zhu L Zhou G Xie B Cui Y. Imbalanced distribution of regulatory T cells and Th17.1 cells in the peripheral blood and BALF of sarcoidosis patients: relationship to disease activity and the fibrotic radiographic phenotype. Front Immunol. (2023) 14:1185443. doi: 10.3389/fimmu.2023.1185443 37520566 PMC10374842

[39] Hata K Yanagihara T Matsubara K Kunimura K Suzuki K Tsubouchi K. Mass cytometry identifies characteristic immune cell subsets in bronchoalveolar lavage fluid from interstitial lung diseases. Front Immunol. (2023) 14:1145814. doi: 10.3389/fimmu.2023.1145814 36949950 PMC10027011

[40] Mendoza N Casas-Recasens S Olvera N Hernandez-Gonzalez F Cruz T Albacar N. Blood immunophenotypes of idiopathic pulmonary fibrosis: Relationship with disease severity and progression. Int J Mol Sci. (2023) 24:13832. doi: 10.3390/ijms241813832 37762135 PMC10531459

[41] Wu Z Chen H Ke S Mo L Qiu M Zhu G. Identifying potential biomarkers of idiopathic pulmonary fibrosis through machine learning analysis. Sci Rep. (2023) 13:16559. doi: 10.1038/s41598-023-43834-z 37783761 PMC10545744

[42] Serezani APM Pascoalino BD Bazzano JMR Vowell KN Tanjore H Taylor CJ. Multiplatform single-cell analysis identifies immune cell types enhanced in pulmonary fibrosis. Am J Respir Cell Mol Biol. (2022) 67:50–60. doi: 10.1165/rcmb.2021-0418OC 35468042 PMC9273229

[43] Dai X Yang Z Zhang W Liu S Zhao Q Liu T. Identification of diagnostic gene biomarkers related to immune infiltration in patients with idiopathic pulmonary fibrosis based on bioinformatics strategies. Front Med. (2022) 9:959010. doi: 10.3389/fmed.2022.959010 PMC9729277 36507532

[44] Unterman A Zhao AY Neumark N Schupp JC Ahangari F Cosme C. Single-cell profiling reveals immune aberrations in progressive idiopathic pulmonary fibrosis. Am J Respir Crit Care Med. (2024). doi: 10.1164/rccm.202306-0979OC PMC11351796 38717443

[45] Moeller A Ask K Warburton D Gauldie J Kolb M. The bleomycin animal model: A useful tool to investigate treatment options for idiopathic pulmonary fibrosis? Int J Biochem Cell Biol. (2008) 40:362–82. doi: 10.1016/j.biocel.2007.08.011 PMC2323681 17936056

[46] Jansen K Cevhertas L Ma S Satitsuksanoa P Akdis M Van De Veen W. Regulatory B Cells, A to Z. Allergy. (2021) 76:2699–715. doi: 10.1111/all.14763 33544905

[47] Rosser EC Mauri C. Regulatory B Cells: Origin, phenotype, and function. Immunity. (2015) 42:607–12. doi: 10.1016/j.immuni.2015.04.005 25902480

[48] Glass MC Glass DR Oliveria J-P Mbiribindi B Esquivel CO Krams SM. Human IL-10-producing B Cells have diverse states that are induced from multiple B cell subsets. Cell Rep. (2022) 39:110728. doi: 10.1016/j.celrep.2022.110728 35443184 PMC9107325

[49] Asai Y Chiba H Nishikiori H Kamekura R Yabe H Kondo S. Aberrant populations of circulating T follicular helper cells and regulatory B Cells underlying idiopathic pulmonary fibrosis. Respir Res. (2019) 20:244. doi: 10.1186/s12931-019-1216-6 31694639 PMC6836348

[50] d’Alessandro M Bergantini L Cameli P Fanetti M Alderighi L Armati M. Immunologic responses to antifibrotic treatment in IPF patients. Int Immunopharmacol. (2021) 95:107525. doi: 10.1016/j.intimp.2021.107525 33714885

[51] Moog MT Hinze C Bormann T Aschenbrenner F Knudsen L DeLuca DS. B Cells are not involved in the regulation of adenoviral TGF-β1– or bleomycin-induced lung fibrosis in mice. J Immunol. (2022) 208:1259–71. doi: 10.4049/jimmunol.2100767 35149532

[52] Ramakrishnan S Bafadhel M. Heterogeneity of IPF exacerbations. Lancet Respir Med. (2022) 10:e3. doi: 10.1016/S2213-2600(21)00459-8 34973212

[53] Karampitsakos T Juan-Guardela BM Tzouvelekis A Herazo-Maya JD. Precision medicine advances in idiopathic pulmonary fibrosis. eBioMedicine. (2023) 95. doi: 10.1016/j.ebiom.2023.104766 PMC10469771 37625268

